# 7-Ketocholesterol Promotes Oxiapoptophagy in Bone Marrow Mesenchymal Stem Cell from Patients with Acute Myeloid Leukemia

**DOI:** 10.3390/cells8050482

**Published:** 2019-05-21

**Authors:** Jessica Liliane Paz, Debora Levy, Beatriz Araujo Oliveira, Thatiana Correia de Melo, Fabio Alessandro de Freitas, Cadiele Oliana Reichert, Alessandro Rodrigues, Juliana Pereira, Sergio Paulo Bydlowski

**Affiliations:** 1 Laboratory of Genetics and Molecular Hematology (LIM31), Department of Hematology, Hospital das Clinicas HCFMUSP, Faculdade de Medicina, Universidade de Sao Paulo, Sao Paulo 05403-000, SP, Brazil; paz.jl@usp.br (J.L.P.); d.levy@hc.fm.usp.br (D.L.); beatriz_oliveira@usp.br (B.A.O.); thatianacmelo@yahoo.com.br (T.C.d.M.); fabio.alessandro@alumni.usp.br (F.A.d.F.); kadielli@hotmail.com (C.O.R.); 2 Departmento de Ciencias Exactas e da Terra, Universidade Federal de Sao Paulo, Diadema 09972-270, SP, Brazil; alessandro.rodrigues@unifesp.br; 3 Center of Innovation and Translational Medicine, Department of Medicine, Faculdade de Medicina, Universidade de Sao Paulo, Sao Paulo 05403-000, SP, Brazil; julianapereira29@hotmail.com; 4 National Institute of Science and Technology for Regenerative Medicine (INCT Regenera), CNPq, Rio de Janeiro 21941-902, Brazil

**Keywords:** oxysterol, 7-KC, mesenchymal stem cell, apoptosis, autophagy, acute myeloid leukemia, bone marrow

## Abstract

7-Ketocholesterol (7-KC) is a cholesterol oxidation product with several biological functions. 7-KC has the capacity to cause cell death depending on the concentration and specific cell type. Mesenchymal stem cells (MSCs) are multipotent cells with the ability to differentiate into various types of cells, such as osteoblasts and adipocytes, among others. MSCs contribute to the development of a suitable niche for hematopoietic stem cells, and are involved in the development of diseases, such as leukemia, to a yet unknown extent. Here, we describe the effect of 7-KC on the death of bone marrow MSCs from patients with acute myeloid leukemia (LMSCs). LMSCs were less susceptible to the death-promoting effect of 7-KC than other cell types. 7-KC exposure triggered the extrinsic pathway of apoptosis with an increase in activated caspase-8 and caspase-3 activity. Mechanisms other than caspase-dependent pathways were involved. 7-KC increased ROS generation by LMSCs, which was related to decreased cell viability. 7-KC also led to disruption of the cytoskeleton of LMSCs, increased the number of cells in S phase, and decreased the number of cells in the G1/S transition. Autophagosome accumulation was also observed. 7-KC downregulated the SHh protein in LMSCs but did not change the expression of SMO. In conclusion, oxiapoptophagy (OXIdative stress + APOPTOsis + autophagy) seems to be activated by 7-KC in LMSCs. More studies are needed to better understand the role of 7-KC in the death of LMSCs and the possible effects on the SHh pathway.

## 1. Introduction

Acute myeloid leukemia (AML) is a malignant disease of the hematopoietic cells characterized by impaired differentiation and uncontrolled clonal expansion of myeloid progenitors/precursors, resulting in bone marrow (BM) failure and impaired normal hematopoiesis [1]. Currently, 40–50% of AML patients can be cured using conventional chemotherapy [2]. However, the incidence of AML increases exponentially with age, and the cure rate is significantly lower in older adults [3]. Although high levels of supportive care have improved patient outcomes, a high cure rate in AML has not yet been achieved [2].

Hematopoietic stem cells (HSCs) reside in the BM niche, a specialized microenvironment that contains complex and diverse stromal cell populations, mainly adipocyte cells and osteocyte [4]. Mesenchymal stem cells (MSCs) are multipotent cells that are also found in the niche and generate most BM stromal cell lineages. MSCs can lose their unspecialized or undifferentiated states and transform into other mesenchymal lineages [5], generating osteoblasts, chondrocytes, fibroblasts, adipocytes, endothelial cells, and myocytes [6]. The self-renewal property is essential for expansion of the stem cell pool during fetal development, as well as maintenance of the stem cell pool throughout the lifespan of the organism [7].

MSCs contribute substantially to the creation of a hematopoietic niche [8,9]; they can secrete several cytokines/growth factors that can modulate the immune response and increase the potential for the expansion and differentiation of host cells [10]. Furthermore, in the BM they provide a niche for the growth, differentiation, and survival of normal and malignant hematopoietic cells, playing an essential role in normal hematopoiesis by regulating HSC proliferation and differentiation [11,12]. Mutations in stromal cells from the tumor microenvironment have been reported to be involved in malignant transformation in breast and colon cancer, as well as myelodysplastic syndromes (MDSs) and AML [13]. Recently, an inherent defect in AML mesenchymal stem cells (LMSCs) was demonstrated to lead to dysfunctional crosstalk between the BM niche and HSCs, potentially resulting in AML initiation or propagation [14]. Specific changes in MSCs have also been demonstrated to initiate leukemia in mice [15,16,17]. In addition, it is well accepted that BM stromal cells, including MSCs, promote leukemia cell resistance to chemotherapy [12].

Cholesterol is essential for cell function and viability; it is a component of the plasma membrane and lipid rafts, and is a precursor of several compounds, including bile acids and steroid hormones [18]. Oxidation and peroxidation are important features of lipid metabolism [19,20]. Cholesterol is also susceptible to cellular oxidation induced by enzymes or reactive oxygen species (ROS), generating several oxidized derivatives [21]. Several oxysterols are biologically active as regulatory molecules and are involved in the regulation of sterol and lipid metabolism, the modulation of signaling pathways, and influencing cell proliferation and differentiation [22]. A number of important roles have been ascribed to oxysterols, such as cholesterol turnover, atherosclerosis, apoptosis, necrosis, inflammation, and immunosuppression [23].

7-Ketocholesterol (7-KC) is an oxysterol that differs from free cholesterol by the addition of a functional ketone group at C7 [24]. It is a biologically active molecule capable of promoting, among numerous cell types, an apoptotic mode of cell death associated with autophagy (oxiapoptophagy) [25,26,27,28,29,30] and characterized by reduced cell proliferation, increased membrane permeability, nucleus condensation, decreased production of nitric oxide, and oxidative damage to the DNA [31].

7-KC has also been shown to induce cell death in MSCs isolated from adipose tissue, as well as cell apoptosis through mitochondrial and nuclear damage [30]. However, the actions of 7-KC in the MSCs of other organs and tissues are not very well-known, and even less so in those affected by disease. Since MSCs are essential for the maintenance of the bone marrow niche and consequently hematopoiesis, this work was designed to evaluate the action of 7-KC in causing cell death in the bone marrow MSCs of patients with AML, including the type of death and possible mechanisms.

## 2. Materials and Methods

### 2.1. Isolation and Characterization of Mesenchymal Stem Cells from Patients with AML (LMSCs)

All subjects gave their informed consent for inclusion before they participated in the study. The study was conducted in accordance with the Declaration of Helsinki, and the protocol was approved by the Ethics Committee of University of Sao Paulo.

Medical School (CAE: 89768418.7.0000.0068). BM was aseptically collected from the iliac crest of five patients with AML at the time of diagnosis [32]. Heparinized BM was washed with PBS and centrifuged for 7 min at 700× *g*. After resuspension and loading on Ficoll gradient (Ficoll-PaqueTM Plus, GE Healthcare, Uppsala, Sweden), the mononuclear cell fraction was collected. Cells were centrifuged for 7 min at 700× *g* and washed with PBS. Finally, LMSCs were resuspended in MSC medium and plated in 75-cm^2^ culture flasks (Santa Cruz Biotechnology, Dallas, TX, USA). The MSC medium consisted of DMEM supplemented with 20% heat-inactivated fetal bovine serum (FBS) (Vitrocell, Waldkirch, Germany) and 1% antibiotics streptomycin (100 µg/mL; Sigma Aldrich, San Luis, MO, USA) and penicillin (100 UI/mL; Sigma Aldrich). After transferring to flasks, the cells were incubated at 37 °C in a 5% CO_2_ atmosphere. Before reaching confluence, cells were detached using a trypsin-EDTA solution (Gibco, Waltham, MA USA) and seeded at a density of 5 × 10^3^ cells/cm^2^. Cells were used for experiments at the 4th to 6th passage.

Cell surface markers for LMSC identification were measured using flow cytometry in a FACSCalibur flow cytometer (BD Biosciences, Franklin Lakes, NJ, USA). After trypsinization and washing with phosphate buffered saline (PBS), approximately 5 × 10^5^ cells were stained for 60 min in the dark with primary monoclonal antibodies against CD29 (CD2004-R-PE), CD44 (MHCD4401-FITC), CD105 (MHCD10504R-PE), CD34 (CD34-581-01-FITC), CD11b (RM2804-3-PE), CD45 (MHCD4504R-PE), CD90 (11-0909-42-FITC), and HLA-DR (11-9956-4-FITC) (all from Invitrogen Life Technologies, Waltham, MA USA). A total of 10,000 events were acquired per acquisition using BD CellQuest Pro software (version 5.1, BD Biosciences). Finally, LMSCs were also characterized by their osteogenic, adipogenic, and chondrogenic differentiation capability in vitro as described elsewhere [33].

### 2.2. Stem Cell Treatments

7-KC was synthesized from cholesterol (Sigma Aldrich) as described elsewhere [34,35]. The purity of 7-KC was determined to be ~98% by GS/MS. For all experiments, a stock solution was prepared in absolute ethanol at a concentration of 10,000 µM. The concentrations used in the experiments were in the range of those described to induce cell death in several cell lines [29]. For the experiments, LMSCs from each donor were plated in 96-well Black Flat Bottom Polystyrene Microplates (Corning, Somerville, MA, USA) at a density of 1.5 × 10^3^ cells/cm^2^ and incubated as described above. Several concentrations of 7-KC (0 to 100 µM, 100 µL final volume) were added to the media and incubated for 24 h. At the end of this period, several experiments were performed in at least duplicate.

### 2.3. Cell Viability Assay

LMSCs were plated at a density of 1.5 × 10^3^ cells/cm^2^ in 96-well Black Flat Bottom Polystyrene Microplates. After 24 h, the cells were pre-treated for 3 h with 20 µM Z-VAD-FMK (BioVision, Milpitas, CA, USA), 10 mM 3-methyladenine (BioVision), or 100 µM necrostatin-1 (ABCAM, Cambridge, UK), or for 4 h with 4 mM *N*-acetyl-l-cysteine (NAC; Eurofarma, SP, Brazil). The media was then changed and 7-KC added at different concentrations for 24 h. LMSCs without pre-treatment were used as controls. Cells were then incubated with 0.1 µg/mL Hoechst 33342 (Molecular Probes, Eugene, OR, USA) and 0.5 μL propidium iodide (PI) (Molecular Probes) for 15 min. The ImageXpress Micro High Content Screening System (Molecular Devices) was used to determine the number of live and dead cells. Nine sites per well and three wells per treatment were acquired. Cell Scoring MetaXpress software (version 5, Molecular Devices) was used to analyze the number of cells and the viability as previously described [36].

### 2.4. Apoptosis and Necrosis

A total of 1.5 × 10^3^ cells/cm^2^ were plated in 96-well Black Flat Bottom Polystyrene Microplates. After 24 h, the cells were incubated with different concentrations of 7-KC (10–100 µM) for another 24 h. The Annexin V: FITC Apoptosis Detection Kit I (BD Biosciences) was used to determine the percentage of apoptotic cells as described by the manufacturer. Briefly, cells were incubated with 0.5 μL FITC Annexin V and 2.0 μL PI (50 µM/mL) and incubated for 15 min at room temperature in the dark. The nuclei were counterstained with 0.1 µg/mL Hoechst 33342 for 10 min. The presence of apoptosis was analyzed within 1 h using an ImageXpress Micro High Content Screening System (Molecular Devices). Five sites per well and two wells per treatment were acquired. The proportion of apoptotic cells was determined using MetaXpress Cell Health software (version 5, Molecular Probes) [36]. Cells stained with Hoechst 33342 were considered to be living cells. An apoptotic process was defined by the presence of Annexin V or Annexin V/PI. A necrotic process was defined by the presence of PI alone.

### 2.5. LC3B Protein Aggregation-Autophagy

Autophagy was detected by measuring the aggregation of LC3B protein coupled to green fluorescence protein (GFP) using the Premo Autophagy Sensor kit (Invitrogen Life Technologies) as described by the manufacturer. Briefly, LMSCs were seeded in 96-well Black Flat Bottom Polystyrene Microplates at 1.5 × 10^3^ per well. The cells were transduced with non-replicating baculoviral vectors expressing LC3B-GFP (component A) or LC3BΔ (G120A)-GFP (component B), which encodes a point mutation in the LC3B gene that prevents cleavage. Sixteen hours after transduction, the cells were treated with 7-KC (30, 50, or 70 μM) and cloroquine diphosphate (30 µM) for 24 h. The appearance of LC3B-GFP aggregates was observed and photographed using an ImageXpress Micro High Content Screening System. Five sites per well and two wells per treatment were acquired. Cell Scoring MetaXpress software was used to analyze the number of cells with aggregation of LC3B-GFP protein.

### 2.6. Detection of Caspase-3/7 Activity

The cells were plated at a density of 1.5 × 10^3^ cells/cm^2^ in 96-well Black Flat Bottom Polystyrene Microplates and treated with different concentrations of 7-KC for 24 h. Caspase-3/7 activity was measured using CellEvent Caspase-3/7 Green (Invitrogen Life Technologies). The nuclei were counterstained with 0.1 µg/mL Hoechst 33342. Fluorogenic substrates were determined using the ImageXpress Micro High Content Screening System. Five sites per well and two wells per treatment were acquired. Caspase-3/7 activity was determined using Cell Scoring MetaXpress software [30].

### 2.7. Detection of Caspase-8

Active caspase-8 was determined using the Vybrant**^®^** FAM Caspase-8 Assay Kit (Molecular Probes, ugene, OR, USA) following the manufacturer’s instructions. Briefly, 1.5 × 10^3^ cells/cm^2^ were plated in 96-well Black Flat Bottom Polystyrene Microplates and treated with different concentrations of 7-KC for 24 h. After treatment, cells were stained with FAM-LETD-FMK. Cell fluorescence was measured using the ImageXpress Micro High Content Screening System. Five sites per well and two wells per treatment were acquired. Caspase-8 was determined using Cell Scoring MetaXpress software.

### 2.8. Cell Cycle Evaluation with Hoechst 33342

After 24 h exposure to 7-KC, cells were incubated with Hoechst 33342 (0.1 µg/mL) for 15 min. The ImageXpress Micro High Content Screening System was used to determine the cell cycle. Five sites per well and two wells per treatment were acquired. Cell Cycle MetaXpress software was used to analyze the different phases of the cell cycle [36].

### 2.9. Measurement of Transmembrane Mitochondrial Potential (ΔΨ)

LMSCs were plated at a density of 1.5 × 10^3^ cells/cm^2^ and treated with 7-KC for 24 h. At the end of the experimental period, treated cells were incubated with 50 nM TMRE (Sigma Aldrich) for 30 min at 37 °C as described by the manufacturer. TMRE is a potentiometric and cationic indicator die that accumulates preferentially in energized mitochondria cells [37]. Nuclei were counterstained with 0.1 µg/mL Hoechst 33342. TMRE fluorescence was determined using ImageXpress Micro High Content Screening System. Five sites per well and two wells per treatment were acquired. Transmembrane mitochondrial potential was determined using Cell Scoring MetaXpress software [30].

### 2.10. ROS Measurement

ROS were determined using the DCFDA/H2DCFDA-Cellular ROS Assay Kit (Abcam, Cambridge, UK) as described by the manufacturer. LMSCs were plated at a density of 1.5 × 10^3^ cells/cm^2^ and treated with 7-KC for 24 h. At the end of the experimental period, treated cells were incubated with 10 µM DCFH-DA in culture media for 45 min. The cells were washed with buffer provided by the kit and suspended in PBS. Intracellular ROS production was determined using ImageXpress Micro High Content Screening System. Five sites per well and two wells per treatment were acquired. ROS was determined using Cell Scoring MetaXpress software.

### 2.11. Detection of Smoothened (SMO) and Sonic Hedgehog (SHh) by Indirect Immunofluorescence

For the indirect immunofluorescence experiments, cells were plated at a density of 1.5 × 10^3^ cells/cm^2^ in 96-well Black Flat Bottom Polystyrene Microplates, treated with 7-KC for 24 h, and fixed in 4% paraformaldehyde (Sigma-Aldrich) for 2 h at 4 °C. After washing with Dulbecco′s Phosphate Buffered Saline (DPBS), the cells were permeabilized with a 0.1% Triton X-100 solution (Sigma-Aldrich) at 4 °C for 15 min, followed by blocking with 5% BSA (Sigma-Aldrich) for 40 min at room temperature. The cells were incubated overnight with the antibody SMO (1:100 dilution; Novus Biologicals, Centennial, CO, USA) or SHh (1:400 dilution; Abcam, Cambridge, UK). The cells were then incubated for 1 h with the anti-rabbit secondary antibody AlexaFluor 488 (1:1000 dilution; Molecular Probes, Eugene, Oregon, EUA). The intensity of the fluorescence of SMO and SHh was determined using the ImageXpress Micro High Content Screening System. Nine sites per well and two wells per treatment were acquired. The cell intensity of fluorescence was determined using Cell Scoring MetaXpress software [38].

### 2.12. Changes in F-Actin Organization

Changes in F-actin organization were investigated using Alexa Fluor 532 phalloidin (Molecular Probes) as previously described [30]. Cells were fixed in a solution of 4% paraformaldehyde for 15 min. After rinsing twice with DPBS, the cells were permeabilized with a 0.1% Triton X-100 solution (Sigma Aldrich) at 4 °C for 10 min, followed by incubation with 3 U/mL phalloidin in DPBS for 2 h. After washing with DPBS, cell nuclei were stained with 300 nM DAPI. The plates were then washed twice with DPBS and analyzed using ImageXpress Micro High Content Screening System. Nine sites per well and two wells per treatment were acquired [30].

### 2.13. Statistical Analysis

Results are expressed as mean ± SEM from at least three independent experiments of each sample. Means were compared using ANOVA followed by the Dunnett post-hoc test in GraphPad Prism (version 8, GraphPad Software, La Jolla, CA, USA). *P*-values ≤0.05 were considered significant.

## 3. Results

### 3.1. Characterization of Bone Marrow Mesenchymal Stem Cells from AML Patients

All five patients were diagnosed with AML not otherwise specified according to the 2016 revision of the World Health Organization classification of myeloid neoplasms and acute leukemia [39]. Two of the patients had AML with maturation; the other three had AML without maturation (acute monoblastic/monocytic leukemia or acute myelomonocytic leukemia). Isolated LMSCs adhered to plastic and had a fibroblast-like morphology. They were characterized as MSCs by the expression of CD29, CD44, CD90, and CD105 and lack of expression of CD34 (present in hematopoietic stem cells), CD11b (present in macrophages), CD45 (present in leukocytes), and HLA-DR. LMSCs were cultivated in specific osteogenic, adipogenic, and chondrogenic differentiation media to evaluate their plasticity and multipotency. Differentiation was confirmed after 21 days (data not shown).

### 3.2. Death of Bone Marrow Mesenchymal Stem Cells from AML Patients after 24 h Incubation with 7-KC

**Figure 1:**
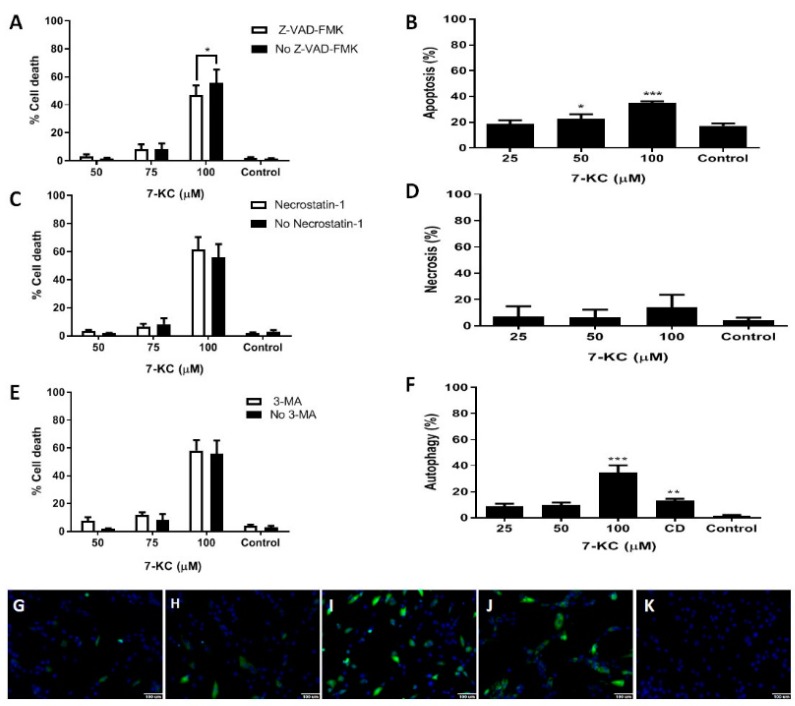
Apoptosis, necrosis, and autophagy in bone marrow-derived mesenchymal stem cells from patients with acute myeloid leukemia after 24 h 7-KC treatment. **A**: Cells were treated with or without Z-VAD-FMK for 3 h followed by incubation with 7-KC for 24 h. Cytotoxicity (apoptosis) was evaluated by Hoechst 33342/propidium iodide staining. **B**: Percentage of apoptotic cells determined by the externalization of phosphatidylserine. **C**: Cells were treated with or without necrostatin-1 for 3 h followed by incubation with 7-KC for 24 h. Cytotoxicity (necrosis) was evaluated by Hoechst 33342/propidium iodide staining. **D**: Percentage of cells with necrosis determined by propidium iodide. **E**: Cells were treated with or without 3-MA for 2 h followed by incubation with 7-KC for 24 h. Cytotoxicity (autophagy) was evaluated by Hoechst 33342/propidium iodide staining. **F**: Percentage of cells with autophagy as evaluated by Premo Autophagy Sensor. CD: chloroquine diphosphate. **G**–**K**: Representative figures of LC3B-green fluorescent protein (GFP) chimera sensing of autophagy (**G**: 25 µM 7-KC; **H**: 50 µM 7-KC; **I**: 100 µM 7-KC; **J**: CD; and **K**: control). Data are mean ± SEM from three independent experiments in duplicate. * *p* < 0.05, ** *p* < 0.01, *** *p* <0.001. Scale bar, 100 µM.

7-KC was tested at 10, 25, 50, and 100 µM for 24 h (data not shown) and found to be cytotoxic for LMSCs in a concentration-dependent manner. The IC50 was 81.1 µM. To determine the role of caspase-dependent programmed cell death, the pan-caspase inhibitor Z-VAD-FMK, was used to selectively inhibit the apoptotic pathway [40]. LMSCs were pre-treated with 100 µM Z-VAD-FMK, followed by incubation with different concentrations of 7-KC (50, 75, or 100 µM) for 24 h. As shown in Figure 1A, incubation with Z-VAD-FMK decreased the cell death promoted by 100 µM 7-KC (*p* < 0.05), indicating the presence of apoptosis. Z-VAD-FMK alone and 7-KC at lower concentrations did not change the proportion of dead cells. Apoptosis was further analyzed using the Annexin V and PI assay, counterstaining the nucleus with Hoechst 33342 for cell localization on an image system (Figure 1B). Fifty or 100 µM 7-KC was able to promote apoptosis (22% and 34% apoptotic cells, respectively).

To determine the caspase-independent programmed necrosis pathway, a specific necroptosis inhibitor was used. Necrostatin-1 (Nec-1) is an in vitro selective allosteric inhibitor of death domain receptor-associated adaptor kinase RIP1 [41]. LMSCs were pre-treated with 100 µM Nec-1, and then incubated with different 7-KC concentrations for 24 h. Incubation with Nec-1 did not change the effect of 100 µM 7-KC on cell death (Figure 1C), showing no sign of necrosis. Experiments with PI with lack of Annexin V staining also confirmed the absence of necrosis (Figure 1D).

Autophagy is a multi-stage biochemical process via which eukaryotic cells die by degrading their own cytoplasm and organelles. In this process, the cellular components are engulfed and then digested in double membrane-bound vacuoles called autophagosomes [42]. 3-Methyladenine (3-MA) is commonly used as an inhibitor of autophagy by blocking autophagosome formation via the inhibition of type III phosphatidylinositol 3-kinase (PI-3K) [36]. LMSCs were pre-treated with 10 mM 3-MA for 4 h, followed by incubation with different concentrations of 7-KC for 24 h. Incubation with 3-MA did not affect 7-KC-promoted cell death at 100 µM (Figure 1E). LMSCs were then treated with 7-KC or chloroquine diphosphate (30 µM) for 24 h and the presence of autophagosomes evaluated by LC3B-GFP staining under fluorescence microscopy (Figure 1G–K). A significant increase (*p* < 0.001) in autophagosomes was observed only in cells treated with 100 µM 7-KC (Figure 1F).

**Figure 2:**
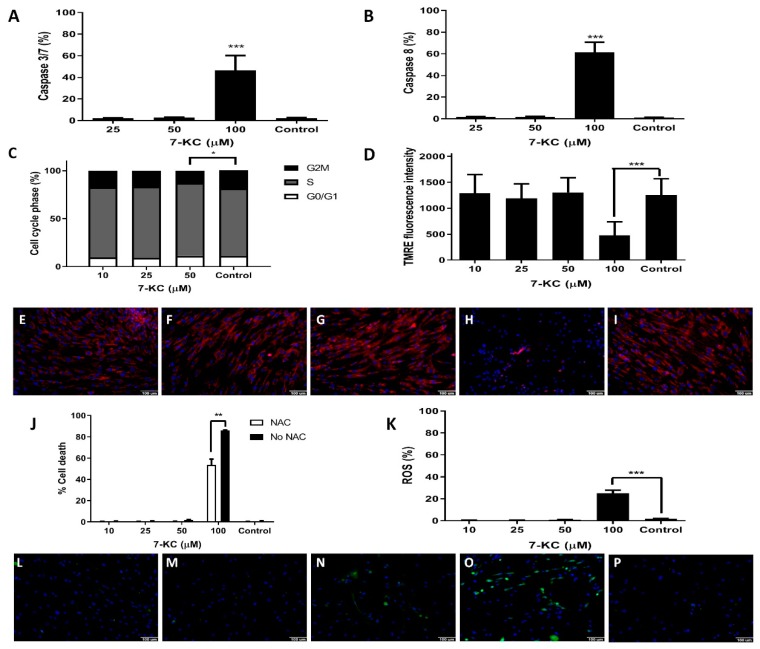
Caspase-3/7 and caspase-8 activities, cell cycle phases, mitochondrial transmembrane potential, and effects of reactive oxygen species (ROS) on bone marrow mesenchymal stem cells from patients with acute myeloid leukemia after 24 h 7-KC treatment. **A**: Percentage of cells with caspase-3/7 activity measured by CellEvent Caspase-3/7 Green. **B**: Percentage of cells with caspase-8 activity determined by the Vybrant FAM Caspase-8 Assay Kit. **C**: Cell cycle phases determined by Hoechst 33342 staining. **D**: Mitochondrial transmembrane potential evaluated by TMRE. **E**–**I**: Representative mitochondrial staining (**E**: 10 µM 7-KC; **F**: 25 µM 7-KC; **G**: 50 µM 7-KC; **H**: 100 µM 7-KC; and **I**: control). **J**: Cell death and relationship with ROS. Cells were treated with or without *N*-acetyl-l-cysteine (NAC) for 4 h following by incubation with 7-KC. Cytotoxicity was evaluated by Hoechst 33342/propidium iodide staining. **K**: Percentage of cells producing ROS as evaluated by DCFH-DA. **L**–**P**: Representative figures of ROS-producing AML mesenchymal stem cells (LMSCs) (**L**: 10 µM 7-KC; **M**: 25 µM 7-KC; **N**: 50 µM 7-KC; **O**: 100 µM 7-KC; and **P**: control). Data are mean ± SEM from three independent experiments in duplicate. * *p* < 0.05, ** *p* < 0.01, *** *p* < 0.001. Scale bar, 100 µM.

To further examine the mechanism underlying the apoptotic process promoted by 7-KC in LMSCs, we investigated caspase-3/7 (Figure 2A) and caspase-8 (Figure 2B) activities. At 100 µM, 7-KC led to an increase in the activities of both caspase-3/7 and caspase-8 (46%, *p* < 0.001 and 61.17%, *p* < 0.001, respectively). No effect was observed at lower concentrations of 7-KC.

### 3.3. Cell Cycle in Bone Marrow Mesenchymal Stem Cells from AML Patients after Treatment with 7-KC

Cell cycle was evaluated by fluorescent intensity of nuclei stained with Hoechst 33342 from LMSCs after incubation with several 7-KC concentrations for 24 h. Figure 2C and supplementary Table S1 show that 50 μM 7-KC induced S-phase cell cycle arrest (*p* < 0.01), followed by the reduction of the number of cells in G2/M phase (*p* < 0.01). No changes were observed with 7-KC at lower concentrations. The experiment was not performed with 100 μM 7-KC by the significant cell death.

### 3.4. Mitochondrial Changes Promoted by 7-KC in Bone Marrow Mesenchymal Stem Cells from AML Patients

Mitochondrial ΔΨ was measured by TMRE fluorescence intensity analysis in LMSCs in the presence of 7-KC at several concentrations. Alterations in mitochondrial ΔΨ were observed only at 100 µM 7-KC (Figure 2D). Mitochondrial morphology was also analyzed (Figure 2E–I). No mitochondrial changes were observed with 10 and 25 µM 7-KC (Figure 2E,F). After incubation with 50 µM, an increasing number of smaller mitochondria with spotted structures were seen (Figure 2G), whereas almost no staining was observed after incubation with 100 µM (Figure 2H).

### 3.5. 7-KC Induces ROS Production

*N*-acetyl-l-cysteine (NAC) is a synthetic precursor of intracellular cysteine and glutathione, and its anti-ROS activity results from its free radical scavenging property either directly via the redox potential of thiols, or indirectly via increasing glutathione levels in the cells [43]. NAC is commonly used to confirm the involvement of ROS in drug-induced apoptosis. As shown in Figure 2J, pre-incubation of LMSCs with NAC decreased 7-KC cytotoxicity at 100 µM (*p* < 0.01), demonstrating the involvement of ROS in LMSC death. The effect of 7-KC on ROS production was also evaluated after 24 h of treatment (Figure 2K). Lower concentrations of 7-KC did not induce ROS production (Figure 2L–N). ROS expression was observed in 25.07% of cells (*p* < 0.001) after treatment with 100 µM 7-KC (Figure 2O).

### 3.6. 7-KC Down-Regulated SHh but Not SMO

**Figure 3:**
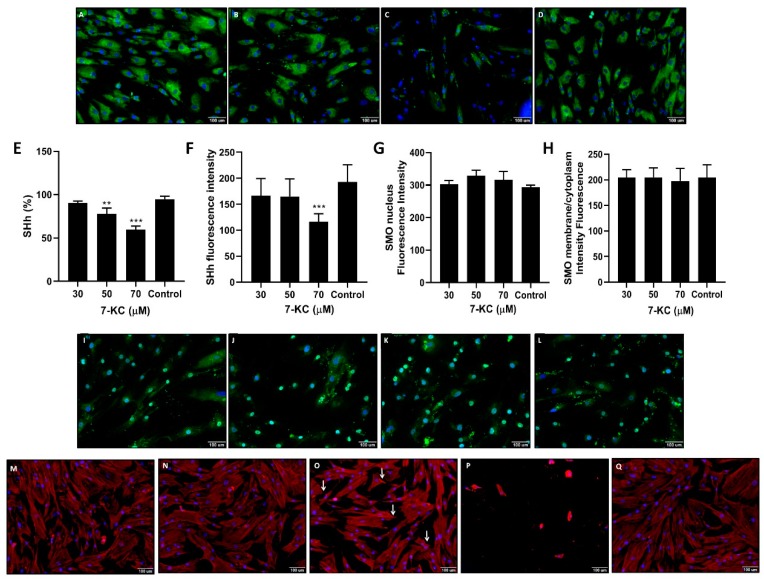
Sonic Hedgehog (SHh), smoothened (SMO), and changes in actin organization in bone marrow mesenchymal stem cells from patients with acute myeloid leukemia after 24 h 7-KC treatment. **A**–**D**: Representative figures of SHh expression measured by immunofluorescence (**A**: 30 µM 7-KC; **B**: 50 µM 7-KC; **C**: 70 µM 7-KC; and **D**: control). **E**: Percentage of cells expressing SHh. **F**: Mean fluorescence intensity of SHh expression. **G**: Mean fluorescence intensity of SMO expression in the nucleus of LMSCs. **H**: Mean fluorescence intensity of SMO expression in the membrane and cytoplasm. **I**–**L**: Representative figures of SMO expression measured by immunofluorescence (**I**: 30 µM 7-KC; **J**: 50 µM 7-KC; **K**: 70 µM 7-KC; and **L**: control). **M**–**Q**: Actin organization evaluated by Alexa Fluor 532 phalloidin staining (**M**: 10 µM; **N**: 25 µM 7-KC; **O**: 50 µM 7-KC; **P**: 100 µM 7-KC; and **Q**: control). White arrow: enhanced fluorescence in the membrane edges. Data are mean ± SEM from three independent experiments in duplicate. ** *p* < 0.01, *** *p* < 0.001. Scale bar, 100 µM.

SHh and SMO protein expression was measured by indirect immunofluorescence. The tested concentrations were below the IC50. The effect of 7-KC on SHh expression was evaluated based on the number of positive cells and the intensity of fluorescence in the cells due to SHh expression (Figure 3A–F). Positive cells decreased with 50 and 70 µM 7-KC (*p* < 0.01 and *p* < 0.001, respectively). The protein expression decreased only at 70 µM 7-KC (*p* < 0.01). The effect of 7-KC on SMO was also evaluated by the intensity of fluorescence in the membrane/cytoplasm and in the nucleus of LMSCs. None of the 7-KC concentrations tested were able to change SMO protein expression (Figure 3G–L).

### 3.7. Changes in LMSC Actin Organization Promoted by 7-KC

Control LMSCs were characterized by the typical fibroblast-like structure with homogeneous distribution of actin fibers (Figure 3Q). Cells treated with 10 or 25 µM of 7-KC (Figure 3M,N) exhibited a cytoplasmic structure modification, with brighter membrane edges. Changes became more evident with increasing concentrations of 7-KC (Figure 3O,P). At 50 µM 7-KC, we observed a diminished cytoplasm area and enhanced fluorescence in the membrane edges (white arrow). At 100 µM 7-KC, we observed a reduced number of cells, and the cytoplasm was shrunken with loss of actin fibers (Figure 3P).

## 4. Discussion

**Scheme 1:**
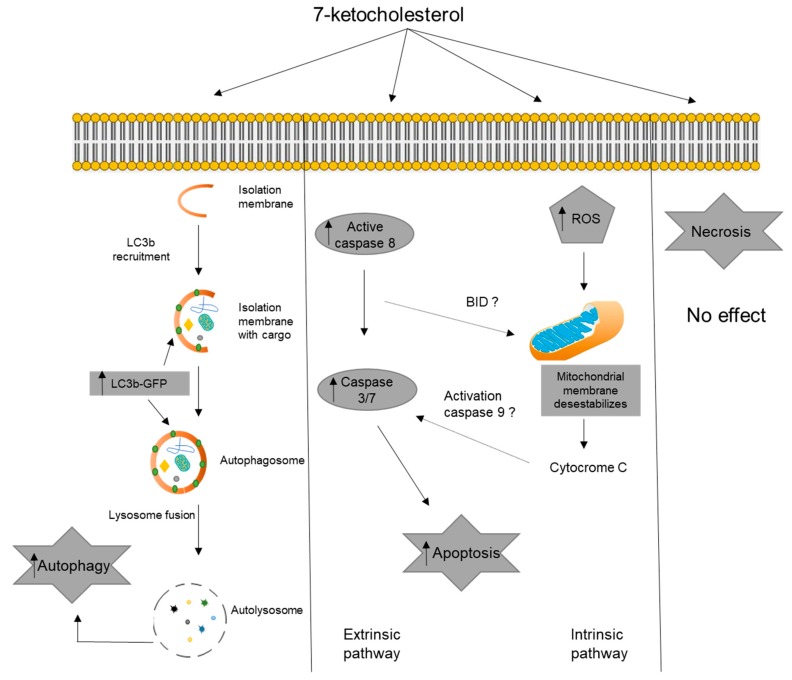
Scheme of cell death promoted by 7-KC in LMSC.

Our finds are summarized in Scheme 1. Human MSCs were isolated from the BM of five patients with AML. They exhibited all of the characteristics of MSCs: adherence to plastic, fibroblast-like morphology, membrane markers CD29+, CD44+, CD90+, and CD105+, lack of membrane markers CD34, CD11b, CD45, and HLA-DR, and the capacity for cell differentiation per The International Society for Cellular Therapy [44]. All LMSC samples were similar in terms of phenotype and differentiation capacity. The main obstacle for MSC isolation and characterization is their low number in fresh tissue, especially BM. Their numbers have been estimated to be approximately 0.01% of nucleated BM cells, declining with age [45]. Corradi et al. [11] were unable to isolate MSCs from the marrow of a substantial fraction (25%) of AML patients, demonstrating the difficulty in isolating this type of cell. In support of their findings, we were unable to obtain LMSCs from the BM of 36% of patients with AML (data not shown).

Ex vivo-expanded MSCs are usually considered to exhibit six biological functions of therapeutic interest: proliferation, multipotency, migration/homing, trophic capacity, immunosuppression, and cell death modulation, often examined independently of one another [46]. Cell death is also characterized by morphological alterations. According to the recommendations of the Nomenclature Committee on Cell Death in 2018 [47], cell death is classified into three types: type I cell death or apoptosis; type II cell death or autophagy; and type III cell death or necrosis.

Most investigations have reported that oxysterols are potent inducers of cell death [48]. Although more than 60 different oxysterols have been described, only some of them are cytotoxic. One of the most studied oxysterols is 7-KC, which has been described as one of the most toxic and predominant element components of oxidized low-density lipoprotein (oxLDL) [49]. Oxysterols such as 7β-hydroxycholesterol and 7-KC are potent inducers of apoptosis in tumor and normal cells [50,51,52,53]. Furthermore, 7-KC induces a particular mode of cell death, oxiapoptophagy [54,55,56].

Reports on the effects of oxysterols on MSCs are relatively scarce, and most are related to the osteogenic effects of some types of oxysterols. Some oxysterols are osteoinductive molecules that can induce the lineage-specific differentiation of cells into osteoblasts [57,58]. Our previous data suggested that 7-KC induces short-term apoptosis in MSCs from adipose tissue, at least in part by direct action on mitochondria [30]. However, other types of oxysterols promote a complex mode of cell death in MSCs, including apoptosis and autophagy, in addition to decreased cell proliferation [36].

Here, we described the early effects of 7-KC on BM MSCs from patients with AML. The cytotoxic effect of 7-KC was tested with the PI method for detecting cell death. The IC50 of 7-KC in LMSCs was 81.1 µM, higher than that described in normal human adipose tissue-derived MSCs (59.54 µM) [30], normal human fibroblasts (47.66 μM) [27], and other cancer cell lines [59]. It is tempting to postulate that LMSCs are less susceptible to the death-promoting effect of 7-KC than other cell types.

Several in vitro studies have described the pro-apoptotic effect of 7-KC in several cell systems [23]. In the extrinsic pathway of apoptosis, caspase-8 and caspase-3/7 are activated. The intrinsic signaling pathway of apoptosis involves a diverse array of non-receptor-mediated stimuli that produce intracellular signals that act directly on targets within the cell. The resulting mitochondrial-initiated events lead to mitochondrial dysfunction and subsequent release of cytochrome c and activation of caspase-9 and caspase-3 [60,61]. Oxysterol-induced apoptosis has been shown to be mediated by both intrinsic mitochondrial pathways and an extrinsic death receptor-dependent pathway [22].

Here, 7-KC exposure triggered the extrinsic pathway of apoptosis as shown by the increase in activated caspase-8. Caspase-8 induces the cleavage and activation of Bid protein, which results in activation of Bax and direct activation of caspase-3 [62]. 7-KC was able to increase the percentage of LMSCs expressing caspase-3 activity. After the signaling pathway is triggered via caspase-3 activation by caspase-8, the death process cannot be stopped, even if negative regulators are involved in the pathway [63].

To further elucidate the 7-KC mode of action in LMSCs, the status of caspase activation was determined using Z-VAD-FMK, a cell-permeant pan-caspase inhibitor [64]. We have demonstrated that Z-VAD-FMK only partially reverses 7-KC-promoting cell death at high concentrations. This suggests that additional mechanisms other than caspase-dependent pathways are involved.

The pro-apoptotic effect of oxysterols has also been associated with overproduction of ROS, changes in intracellular calcium levels, or modification of the mitochondrial membrane associated with the intrinsic pathway [4]. 7-KC is known to induce O^2−^ overproduction [54] and has been shown to activate monocytes/macrophages, resulting in hydrogen peroxide (H_2_O_2_) and superoxide anion (O^2−^) overproduction, lipid peroxidation, plasma membrane alteration, organelle dysfunction, DNA damage, and cytokine secretion, leading to cell death [25].

7-KC increased ROS generation by LMSCs. This increase was related to the decrease in cell viability, as the death of LMSCs was partially reversed in the presence of NAC, a ROS inhibitor. Mitochondria are widely accepted to play a key role in the regulation of apoptosis. Changes in the mitochondrial transmembrane potential in response to various stimuli lead to ROS production and mitochondrial membrane permeabilization (MMP) [23], causing a loss of mitochondrial membrane potential (ΔΨm), with consequent release of pro-apoptotic proteins [40]. In our study, the decrease in ΔΨm in LMSCs in response to 7-KC is evidence of mitochondrial dysfunction.

Although the 7-KC impact mitochondria, their effect on other organelles such as lysosomes could be responsible for the observed effects. The autophagy-lysosomal pathway is a degradation process of dysfunctional organelles that has been associated with several pathophysiological processes [65,66]. The decrease of lysosomal function, including the autophagy-lysosomal pathway, leads to acceleration of ROS generation. Although we did not have investigated the mechanisms of ROS generation, the above observations may further support the interpretation of increased ROS generation promoted by 7-KC in LMSCs.

Components of the cytoskeletal network, such as the actin microfilament meshwork or intermediate filaments, are known targets of oxidative and thiol-depleting agents that induce cell injury [67]. Treatment with oxysterols has been described to cause progressive disruption of actin microfilaments and to induce cell detachment in several cell lines [30,36,67,68,69]. We have also observed that higher concentrations of 7-KC lead to gradual changes in the morphology of LMSCs, with disruption of the cytoskeleton.

A number of studies have shown that oxysterols affect the cell proliferation capacity and cell cycle. HepG2 cells accumulate in G2/M phase upon incubation with 25-hydroxycholesterol [70]. Similar observations have been made in other cell types [71,72,73], but 7-KC does not change the cell cycle in adipose tissue MSCs [36]. Our present data show that the anti-proliferative effects of 7-KC occur in LMSCs through blockage at S phase. We measured an increase in the number of cells in S phase and a decrease in the number of cells in the G1/S transition. The mechanisms responsible for these effects in LMSCs and other cell types remain to be investigated.

It is clear that, despite autophagy being considered a cell survival mechanism, its excessive increase, in certain conditions, may lead to a caspase-independent non-apoptotic type of cell death (type II cell death) [74]. Autophagy plays a consistent role in the modulation of cell proliferation, differentiation, and stemness in a wide variety of cell types, including MSCs [75]. In addition, autophagy has been reported to play an important role in the maintenance and survival of cancer stem cells or tumor-initiating cells in breast and pancreatic carcinomas [76]. Oxidized lipids have been reported to stimulate autophagy in advanced human atherosclerotic plaques and cultured vascular smooth muscle cells [77]. 7-KC also regulates the expression of autophagic genes [78] and has been shown to trigger autophagy in vascular smooth muscle cells and coronary arterial myocytes [79]. Here, we described the accumulation of autophagosomes using fluorescence microscopy with the LC3B-GFP sensor in the presence of high concentrations of 7-KC. In our model system, inhibition of autophagy by the administration of 3-MA in LMSCs did not change the percentage of dead cells after treatment with 7-KC. 3-MA produces a metabolic effect that is not related to the inhibition of autophagy, raising questions about its specificity as an autophagy inhibitor [79]. Therefore, more studies are needed to better understand autophagy in LMSCs after treatment with 7-KC.

Programmed necrosis, also called necroptosis (type III cell death), is a caspase-independent RIP3-mediated form of cell death that was recently identified as a novel mechanism of cell death [80]. Activation of RIP3 is regulated by the kinase RIP1 [81], a key player in the modulation of cell fate in response to different stimuli [82]. Here, we showed, using necrostatin-1, a blocker of both RIPK1 and indoleamine 2,3-dioxygenase (IDO) [83], that necroptosis is not the type of cell death caused by 7-KC.

The Hedgehog (Hh) signaling pathway plays an important role in adult stem cell function and/or progenitor cells in several tissues [84]. Hh signaling occurs through interaction of the Hh ligand with its receptors Ptc and SMO. In the absence of Hh, Ptc inhibits SMO and, through suppression of Gli represses downstream gene expression [80]. When Hh is present, it binds to Ptc and releases SMO, which activates the Hh signaling pathway [66]. Mutations in Hh pathway components, including Ptc1 and SMO, that lead to pathway activation have been linked to basal cell carcinoma and medulloblastoma [85]. The constitutively activated SHh signaling pathway has been shown to be important for growth or progression of liver, lung, stomach, breast, leukemia, and esophageal cancers [85,86]. Hh signals play a critical role in the survival and expansion of cancer stem cells in hematological malignancies [87].

We evaluated the effects of subtoxic doses (70–30 μM) of 7-KC on the SHh pathway and demonstrated that 7-KC is able to downregulate the SHh protein in LMSCs. However, 24 h incubation with 7-KC did not change the expression of SMO. Our previous results with a breast cancer cell line showed no alteration in SHh expression as promoted by different oxysterols; however, 7-KC and cholestan-3α-5β-6α-triol were able to increase the expression of SMO in the nucleus [38]. Selective inhibition of AML cell growth by a SHh pathway antagonist has been shown, with no interference in the growth of normal stem cells [88].

There are some limitations in this study. One is the high concentrations of 7-KC used; in most cases, effects were observed only at the concentration of 100 µM, higher than the IC50 (81 µM). 7-KC studies are usually made with doses much higher than those found physiologically. Even the concentration of 7-KC found in oxidized LDLs is lower than that commonly used in experimental studies. Thus, the biological relevance of 7-KC at high concentrations lies in the perspective of use as a chemotherapeutic adjuvant. Also, activation of caspase 9 has not been studied; nevertheless, other mechanisms participating in this pathway were described, such as mitochondrial alterations and the presence of ROS. Finally, another limitation is the lack of time course experiments, because some effects of 7-KC in low concentration could potentially cause some physiological rather than pharmacological effects after longer periods of time.

In conclusion, taken together, our data demonstrate that oxiapoptophagy, including ROS overproduction, apoptosis, and autophagy, could be a particular type of cell death activated by 7-KC in MSCs from patients with AML. This is the first report showing that oxiapoptophagy can occur in cells other than nerve cells. Although many more studies are needed to better understand the role of oxiapoptophagy caused by 7-KC in LMSCs, it is tempting to postulate that 7-KC, present in oxidized LDL or acting as a pharmacological agent, could modulate leukemic cell proliferation and death by acting in LMSCs.

## Supplementary Materials

The following are available online at https://www.mdpi.com/2073-4409/8/5/482/s1, Table S1. Cell cycle phases determined by Hoechst 33342 staining after 24 h incubation with 7-KC.
