# Supplementary material for: 7-Ketocholesterol Promotes Oxiapoptophagy in Bone Marrow Mesenchymal Stem Cell from Patients with Acute Myeloid Leukemia
Source: Cells. 2019 May 21;8(5):482. doi: 10.3390/cells8050482 (PMC6562391; doi:10.3390/cells8050482)
Supplement: Supplementary file 1 [file cells-08-00482-s001.pdf]

Table S1. Cell cycle phases determined by Hoechst 33342 staining after 24 h incubation with 7-KC

7-KC	G0/G1	S	G2/M
**10 µM**	9,78 ± 2,49	71,55 ± 3,75	18,67 ± 2,70
**25 µM**	9,08 ± 2,23	72,16 ± 2,78	18,75 ± 1,92
**50 µM**	11,13 ± 2,72	76,08 ± 2,86 *	12,79 ± 1,96 *
**Control**	11,01 ± 2,39	70,33 ± 2,95	18,66 ± 0,75

Data are mean ± SEM from three independent experiments in duplicate. * $p<0.05$.
